# Spatiotemporal Determinants of Urban Leptospirosis Transmission: Four-Year Prospective Cohort Study of Slum Residents in Brazil

**DOI:** 10.1371/journal.pntd.0004275

**Published:** 2016-01-15

**Authors:** José E. Hagan, Paula Moraga, Federico Costa, Nicolas Capian, Guilherme S. Ribeiro, Elsio A. Wunder, Ridalva D. M. Felzemburgh, Renato B. Reis, Nivison Nery, Francisco S. Santana, Deborah Fraga, Balbino L. dos Santos, Andréia C. Santos, Adriano Queiroz, Wagner Tassinari, Marilia S. Carvalho, Mitermayer G. Reis, Peter J. Diggle, Albert I. Ko

**Affiliations:** 1 Department of Epidemiology of Microbial Diseases, School of Public Health, Yale University, New Haven, Connecticut, United States of America; 2 Division of Medicine, Lancaster University, Lancaster, United Kingdom; 3 Centro de Pesquisas Gonçalo Moniz, Fundação Oswaldo Cruz, Ministério da Saúde, Salvador, Brazil; 4 Instituto de Saude Coletiva, Federal University of Bahia, Salvador, Brazil; 5 Escola Nacional da Saúde Pública, Fundação Oswaldo Cruz, Ministério da Saúde, Rio de Janeiro, Brazil; University of Queensland School of Veterinary Science, AUSTRALIA

## Abstract

**Background:**

Rat-borne leptospirosis is an emerging zoonotic disease in urban slum settlements for which there are no adequate control measures. The challenge in elucidating risk factors and informing approaches for prevention is the complex and heterogeneous environment within slums, which vary at fine spatial scales and influence transmission of the bacterial agent.

**Methodology/Principal Findings:**

We performed a prospective study of 2,003 slum residents in the city of Salvador, Brazil during a four-year period (2003–2007) and used a spatiotemporal modelling approach to delineate the dynamics of leptospiral transmission. Household interviews and Geographical Information System surveys were performed annually to evaluate risk exposures and environmental transmission sources. We completed annual serosurveys to ascertain leptospiral infection based on serological evidence. Among the 1,730 (86%) individuals who completed at least one year of follow-up, the infection rate was 35.4 (95% CI, 30.7–40.6) per 1,000 annual follow-up events. Male gender, illiteracy, and age were independently associated with infection risk. Environmental risk factors included rat infestation (OR 1.46, 95% CI, 1.00–2.16), contact with mud (OR 1.57, 95% CI 1.17–2.17) and lower household elevation (OR 0.92 per 10m increase in elevation, 95% CI 0.82–1.04). The spatial distribution of infection risk was highly heterogeneous and varied across small scales. Fixed effects in the spatiotemporal model accounted for the majority of the spatial variation in risk, but there was a significant residual component that was best explained by the spatial random effect. Although infection risk varied between years, the spatial distribution of risk associated with fixed and random effects did not vary temporally. Specific “hot-spots” consistently had higher transmission risk during study years.

**Conclusions/Significance:**

The risk for leptospiral infection in urban slums is determined in large part by structural features, both social and environmental. Our findings indicate that topographic factors such as household elevation and inadequate drainage increase risk by promoting contact with mud and suggest that the soil-water interface serves as the environmental reservoir for spillover transmission. The use of a spatiotemporal approach allowed the identification of geographic outliers with unexplained risk patterns. This approach, in addition to guiding targeted community-based interventions and identifying new hypotheses, may have general applicability towards addressing environmentally-transmitted diseases that have emerged in complex urban slum settings.

## Author Summary

Leptospirosis is a rat-borne infectious disease that occurs worldwide, predominantly among vulnerable populations, such as urban slum communities with poor sanitation infrastructure. However, urban slums are complex local settings, where transmission of the disease varies over space and time, and the factors that influence this risk difference are unknown. An improved understanding of the environmental and social factors that modify the risk of this infection is needed in order to guide interventions to reduce the disease burden. We recruited a cohort of 2003 community residents of a high- risk urban slum in Salvador, Brazil. We followed them for a four-year period to understand yearly variation in individual and spatial risk factors for infection using spatiotemporal statistical modeling techniques. Our findings suggest that environmental factors related to topology such as household elevation and inadequate sewage drainage systems increase the risk of transmission in the slum microenvironment by promoting contact with mud contaminated with the pathogenic leptospiral bacteria, and that individual characteristics such as age and gender increase risk through behaviors that lead to increased exposures to a contaminated environment. Through this technique, we also identified local geographic areas where the risks are not well explained by these factors. This will help generate new hypotheses and identify intervention strategies for targeted prevention of leptospirosis in urban slum populations.

## Introduction

Leptospirosis is a leading zoonotic cause of morbidity and mortality, and is estimated to cause one million cases and more than 50,000 deaths each year, at a cost of over 2.90 million DALYs lost per year [1,2]. The disease has traditionally been associated with occupational exposures and rural-based subsistence farming settings [3,4]. However, it has emerged as an important urban health problem in the developing world due to the rapid and disorganized expansion of urban centers, which in turn has created the ecological conditions for rat-borne transmission [4,5]. At present, more than one billion of the world’s inhabitants live in slum settlements. In these settings, large epidemics of leptospirosis have increasingly been reported [6–8]. The infection is caused by a spirochetal bacterium from the genus *Leptospira*. It produces clinical manifestations that range from asymptomatic or mild febrile illness to severe disease [9,10]. Fatality rates for severe disease forms, such as Weil’s disease and pulmonary hemorrhage syndrome, are higher than 10% and 50%, respectively [4,11]. Infections in the urban setting are largely due to a single serogroup, *L*. *interrogans* serogroup Icterohaemorrhagiae, which is acquired during contact with soil or water contaminated with urine of the rat reservoir from which the pathogen is shed [5,6,12–14].

Urban epidemics of leptospirosis are associated with heavy rainfall events. They predominantly affect slum inhabitants due to environmental conditions of inadequate sanitation and drainage infrastructure, and heavy infestation by rodent reservoirs [3,5,15–18]. Previous epidemiological studies of leptospirosis have identified the occurrence of severe cases and infections in association with specific sanitation deficiencies in slums, such as household location in proximity to open sewers and accumulated refuse, in flood-risk areas, and in areas infested by rats, such as *Rattus norvegicus*, which persistently shed leptospires once infected, and are a particularly important reservoir in the urban setting [12,19–23]. Although high-risk urban areas for leptospirosis transmission are generally characterized by low social status and poor sanitation infrastructure, previous studies have shown that they are also highly heterogeneous, with wide spatial variability in social and environmental characteristics that are associated with risk for transmission of *Leptospira* [12,21]. In addition, leptospirosis incidence may vary temporally due to local variability in rainfall, humidity and temperature; however, no studies have evaluated the temporal influence of *Leptospira* transmission in a prospective community-based setting. Finally, annual transmission intensity may be further influenced by unmeasurable factors such as informal interventions by residents that either reduce rodent density or modify drainage patterns, which in turn, influence the frequency and spatial distribution of flooding events.

Spatial variation of leptospirosis risk across the study area may reflect a similar spatial distribution of micro-environmental differences that increase the risk of local environmental contamination with *Leptospira*, or human contact with potentially contaminated environmental compartments. These potential spatial differences may include environmental features such as vegetation, drainage, soil characteristics and rodent reservoir habitability. Likewise, identification of small-scale areas whose risk for transmission is significantly higher or lower than expected, based on measured characteristics, allows further investigation to develop novel hypotheses about leptospirosis transmission risk and identify targets for further study or intervention.

The aim of this study, therefore, was to perform rigorous prospective examination of the risk factors for leptospiral transmission in a high-risk urban slum community in Brazil, accounting for spatial and temporal heterogeneity over and above that attributable to measured risk factors. Herein, we describe findings from four years of prospective study using annual serological and household risk factor surveys. We fit a spatiotemporal multivariable model with both fixed and random effects to identify risk factors and to quantify their effect on *Leptospira* infection. We also examined the spatiotemporal distribution of the random effects, to assess whether the unexplained variation in the pattern of infection exhibits spatiotemporal structure that would require further investigation. Thus, a spatiotemporal approach provides an improved understanding of the underlying epidemiology and the drivers of leptospirosis transmission. It can also identify potential opportunities for effective intervention and control strategies in this and similar slum communities.

## Methods

### Study site and recruitment of the community cohort

**Fig 1:**
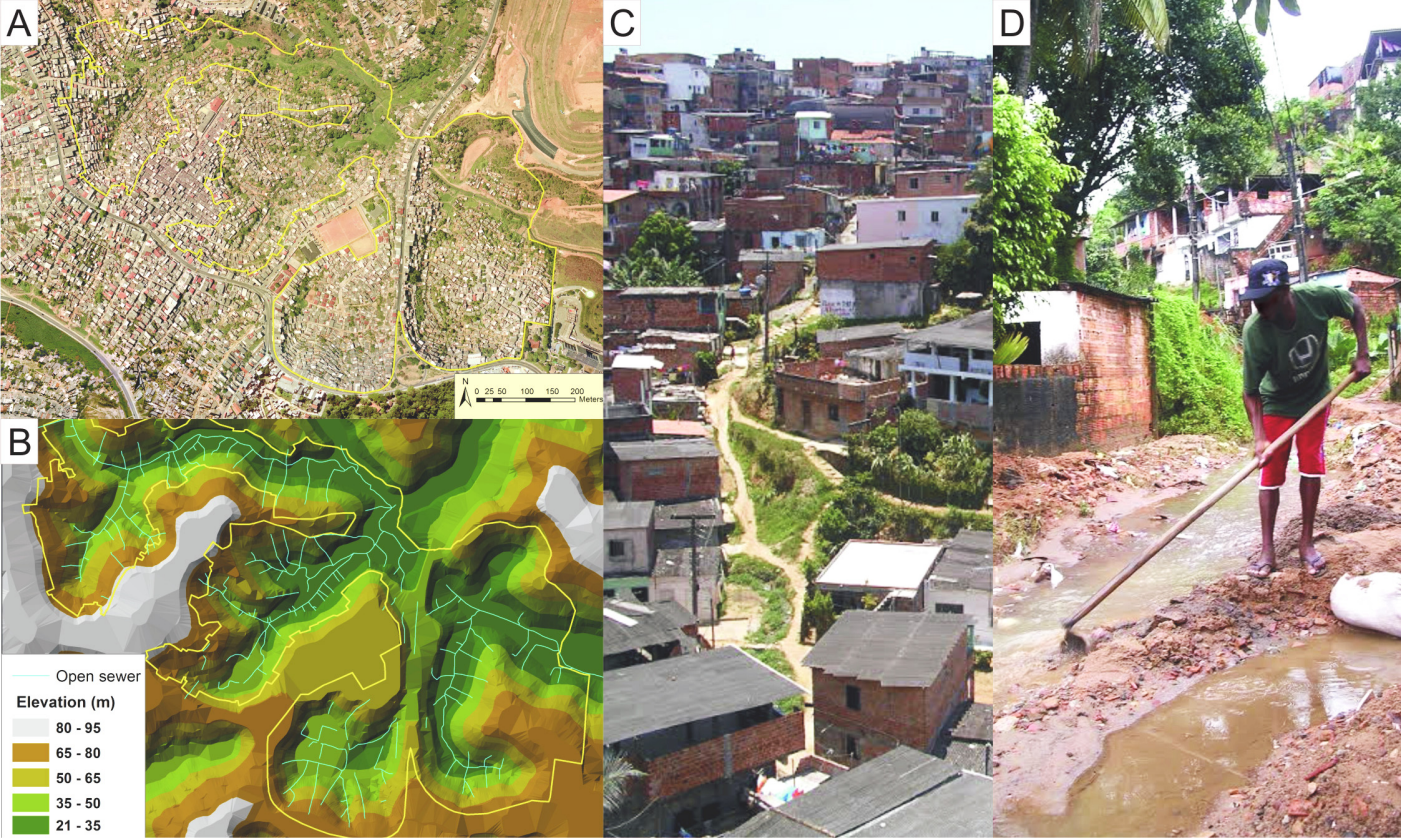
Urban slum community site in the city of Salvador, Brazil. A) An aerial photograph of the study site shows the boundaries of the study site. B) A topographic map of the study site for which open sewer drainage systems have been overlaid. C) A representative photograph demonstrating the social and environmental gradient within the Pau da Lima community. D) Resident in contact with water and mud when cleaning an open sewer.

The prospective cohort study was conducted in the community of Pau da Lima (13°32’53.47” S; 38°43’51.10” W). This slum (*favela*) settlement (Fig 1) is situated in the periphery of Salvador (population, 2,892,625 inhabitants), Brazil [24]. The study site, which has been previously described,[21] has conditions of poverty, land use and climate that are similar to other slum settlements in Brazil and tropical regions in the developing world. In 2003, a study census identified 14,122 inhabitants residing in 3,689 households within the four-valley site with area 0.46 Km^2^. The majority (85%) of inhabitants were squatters who did not have legal title to their domiciles. Median household per capita income was US$1.30 per day. The mean annual incidence of hospitalized leptospirosis at the site was 57.8 cases per 100,000 population between 1996 and 2002 [21]. A one-year seroincidence study of 2,003 residents identified a *Leptospira* infection rate of 37.8 per 1,000 person-years at the study site [21].

A sample of 684 (18% of 3,689) households of all inhabited domiciles was selected using a computer-based random number generator. The sample size of this cohort was informed by seroprevalence surveys [12] and by case-control investigations [19], which found that the frequency of identified risk exposures for anti-*Leptospira* antibodies and leptospirosis is between 20–40% among community individuals. Based on these data, the study was powered to detect a risk ratio of at least 2.0 for potential risk exposures. All subjects aged five years or more who slept three or more nights per week in the sampled households were eligible for enrolment in the cohort study. Subjects were enrolled between February 2003 and May 2004, according to written informed consent procedures approved by the Institutional Review Boards of the Oswaldo Cruz Foundation and Brazilian National Commission for Ethics in Research, Brazilian Ministry of Health, Weill Medical College of Cornell University, and Yale University School of Public Health.

### Prospective ascertainment of risk exposures

During cohort enrollment and annually thereafter during the three subsequent seasonal periods of heavy rainfall and leptospirosis epidemics (May-July) from 2005 to 2007, the study team of 10–15 community health workers, nurses and physicians visited households to interview subjects and administer standardized questionnaires. Interviewers were trained on the study tool and interviewing techniques, and pilot questionnaires were administered in the community prior to initiating data collection. Information was obtained on demographic and socioeconomic indicators, health seeking, employment and occupation, exposures to sources of environmental contamination and presence of potential reservoirs in the household and workplace. We collected information on ethnicity by self-reporting, which is used in Brazil as a marker of socioeconomic status [25,26]. The head-of-household, defined as the member who earned the highest monthly income, was interviewed to determine sources and amounts of income for the household. The study team evaluated literacy according to the ability to read standardized sentences and interpret their meaning. Informal work was defined as income-generating activities for which the subject did not have legal working documents. Exposures to contaminated environment were evaluated by eliciting the subjects’ responses on contact with mud, floodwater, garbage, or sewage during the seasonal period of heavy rainfall. Subjects were asked to report the highest number of rats sighted within the household property and workplace site in the preceding one-month period. The study team surveyed the area within 10 meters of the household to determine the presence of dogs, cats, chickens and vegetation. In addition, the study team surveyed the study site to map the location of open sewage and rainwater drainage systems, identify sites of open accumulated refuse, and measure the area of these deposits. Geographic Information Systems (GIS) were used to obtain tridimensional distance from subject households to the nearest open drainage systems and accumulated refuse, as well as household elevation [12].

### Serologic evaluation of leptospiral infection

The study team collected blood samples from participants during household visits at cohort enrollment and once a year during the seasonal period of low rainfall (November-February), which corresponds to the inter-epidemic period for leptospirosis. The microscopic agglutination test (MAT) was performed on sera to determine titers of agglutinating antibodies against pathogenic *Leptospira*. A panel of five reference strains (WHO Collaborative Laboratory for Leptospirosis, Royal Tropical Institute, Holland) and two clinical isolates [21] was used, which included *L*. *interrogans* serovars Autumnalis, Canicola and Copenhageni, *L*. *borgspetersenii* serovar Ballum, and *L*. *kirschneri* serovar Grippotyphosa. During serologic confirmation of leptospirosis cases [5] and infection in studies performed in Salvador [12,21]this serovar panel demonstrated equivalent performance, to that of the WHO-recommended panel of 16 reference serovars [11]. Screening was performed with serum dilutions of 1:25, 1:50, and 1:100. When agglutination was observed at a dilution of 1:100, the sample was titrated to determine highest agglutination titer. The study outcome of leptospiral infection was defined as seroconversion, an MAT titer increase from negative to ≥1:50, or a four-fold increase in titer between sequential paired samples from cohort subjects. As part of quality control procedures, MAT testing was repeated to confirm all identified infections.

### Statistical modeling

Rates and 95% confidence intervals were estimated based on the number of infections that occurred among cohort subjects during each follow-up event, defined as two sequential annual serosurveys. A mixed effects model was constructed to explain the spatiotemporal variation in leptospiral infection. We first investigated the relationship between infection risk and each potential explanatory variable in turn, using generalized estimating equations with an unstructured working correlation matrix to account for temporal correlation due to repeated measurements, but ignoring spatial correlation [27]. We assessed whether continuous explanatory variables could be assumed to have a linear relationship (on the log-odds scale) with leptospiral infection by fitting a generalized additive model (GAM) [28] and examining the shape of the fitted smooth function. There were no missing values for any of the analyzed variables.

Variables were selected for the spatiotemporal mixed model within eight groups of factors related to: demographic and social status; self-reported prior history of hospitalization for leptospirosis; occupational exposures; household environment; household-related behavior and activities; household reservoirs; occupational behavior and activities; and occupational reservoirs. Within each group, we fitted a GAM using as covariates all of the categorical variables, non-linear effects of the continuous variables, and interactions. We then followed a backward elimination selection process until the minimum value of the modified Akaike Information Criterion (AIC) was achieved [29]. These eight groups of variables were then merged, and the same backward elimination process was followed until it was no longer possible to reduce AIC by elimination of any of the remaining variables. Non-linear effects of continuous variables were accommodated by fitting simple spline models based on the functional form of the components of the GAM model.

Finally, the selected covariates were included as fixed effects into a model that additionally included random effects to account for unexplained spatial and/or temporal variation. The structure of this unexplained variation aids the identification of anomalous areas of high or low risk. Spatiotemporal random effects vary for each location and time but are common to all individuals living in the same household at a given time. We also considered a model with an additional random effect to model unexplained variation in the inherent susceptibility to leptospiral infection between individuals at the same location and time. The models were fitted using the Stochastic Partial Differential Equations (SPDE) approach [30] and Integrated Nested Laplace Approximation (INLA) method [31]. The models were compared using the deviance information criterion (DIC) [32]. We found that the model that included both spatio-temporal and individual-level random effects produced lower DIC values and we therefore used this as the preferred model.

Choropleth maps were constructed to represent the spatiotemporal distribution of infection risk, which included the random component of that risk as described by our multivariable mixed effects model for log odds: $\log\;\left(\frac{p_{ij}}{1-p_{ij}}\right)=z_{ij}\beta +S(x_{i},j)+u_{i}$, where for individual *i* at location *x*_*i*_ and time *j* = 1, 2, 3, 4, *p*_*ij*_ represents the probability of infection, *z*_*ij*_ denotes the vector of covariates, *β* represents the coefficient factor, *S*(*x*_*i*_,*j*) are spatio-temporal random effects and *u*_*i*_ are uncorrelated individual-level random effects.

## Results

The baseline community census identified 14,122 inhabitants of the Pau da Lima study site, of whom 12,651 (90%) were eligible to participate in the cohort [12]. We randomly selected 684 households (18.5%) and approached 2,419 eligible residents (19%) for enrollment, among which 2,003 (83%) consented to participate [21]. Differences between selected vs. non-selected and recruited vs. non-recruited individuals have been described in a previous publication [21]. Over the entire period, data from at least one complete year of follow-up were available from 1,730 (86%) individuals. A total of 1,127 (56%) individuals completed the four follow-up protocol (S1 Table). Compared to participants who completed all four events, those who completed between one and three follow up events were more likely to be male (47% vs. 42%, p = 0.022), and had similar daily per-capita income (median 0.79 dollars per day, interquartile range 0.28–1.26, vs. 0.79 dollars per day, 0.30–1.39, p = 0.248). Change of residence to a household outside the study site was the major cause of loss to follow-up, accounting for 67% of the lost-to-follow-up subjects.

**Fig 2:**
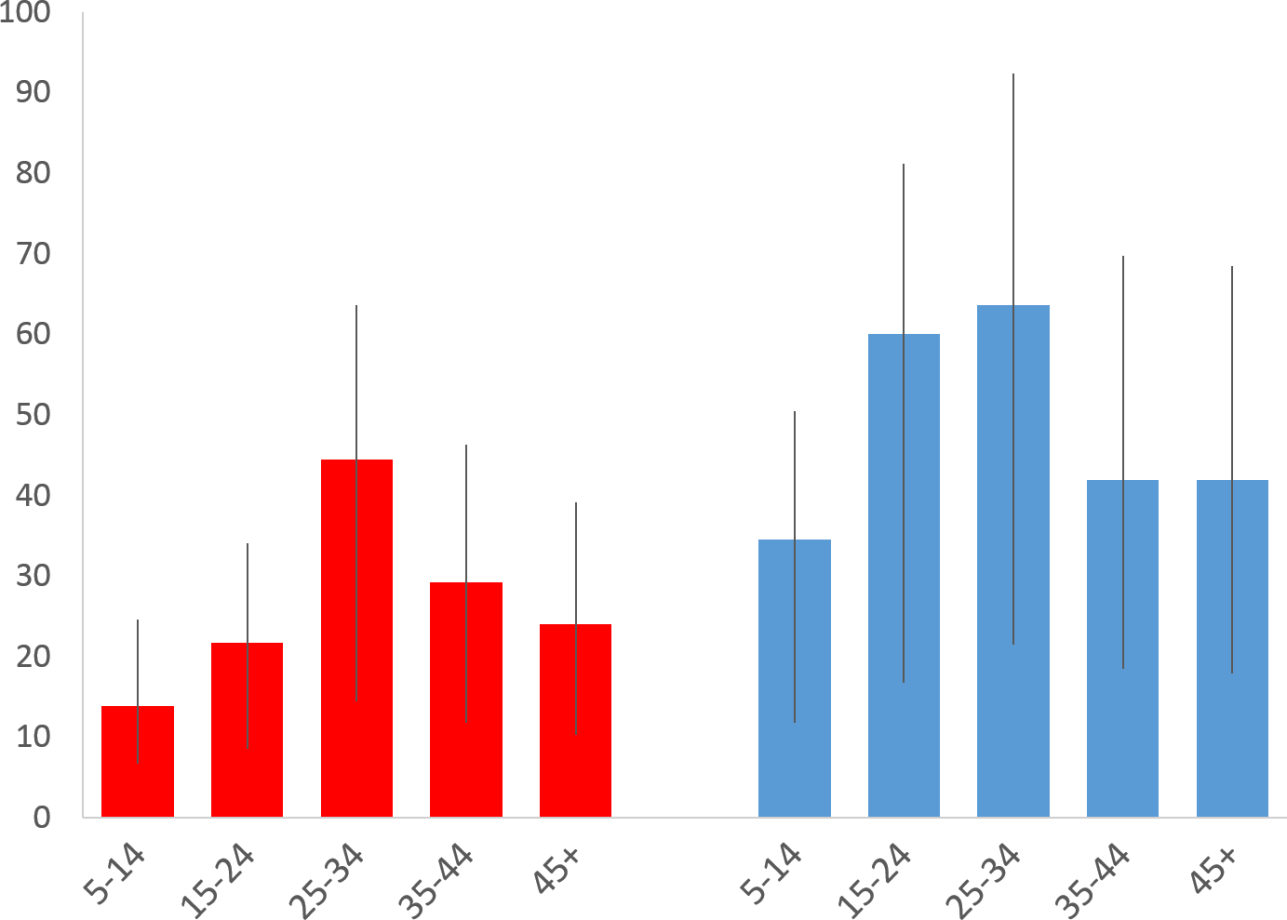
Leptospiral infection rate according to age group and gender. Rates are shown as infections per 1,000 annual follow-up events. Red bars: females. Blue bars: males. Whiskers: 95% CI.

Overall, among 1,730 individuals with at least one complete annual follow-up during the study period, we identified serologic evidence for 199 leptospiral infections among 177 individuals. *L*. *interrogans* serogroup Icterohaemorrhagiae was identified as the presumptive infectious serogroup based on agglutination titers in 178 (90%) of the infections. Other infections were identified as Ballum (9, 4%), Autumnalis (4, 2%), Canicola (2, 1%), Grippotyphosa (2, 1%), and mixed infections including Icterohaemorrhagiae and at least one other serogroup (4, 2%). In 21 of 22 cases where a second or third infection was detected in the same individual, the highest MAT titers were directed against serogroup Icterohaemorrhagiae for both initial and subsequent infections. The overall infection rate was 35.4 (95% CI, 30.7–40.6) per 1,000 annual follow up events for the cohort. The infection rate for the 1,127 subjects who completed the four-year follow-up period was 36.4 (95% CI, 31.1–42.3) infections per 1,000 follow-up events. Among the 248, 195 and 160 subjects who completed only one, two or three follow-up years, respectively, the infection rates were 40.3 (20.7–71.5), 38.5 (22.5–61.8), and 20.8 (10.7–37.0) infections per 1,000 follow-up events. These were not significantly different from the rate for individuals who completed four-year follow-up (P value = 0.335) (S1 Table). Infection rates were higher among males (48.3 infections per 1,000, 95% CI 40.0–57.7, vs. 25.9, 20.8–31.9), and among age groups with 15–24 (39.1, 29.8–50.4) and 25–34 years (52.0, 39.2–67.6) (Fig 2 and S2 Table).

Crude univariable analysis identified characteristics among residents and the slum microenvironment that significantly modified their risk of infection (Table 1). In addition to adult age groups and males, individuals with lower social status (functional illiteracy, and informal employment without contract and benefits) were more likely to acquire infection during follow-up. In addition, a history of previous hospitalization for leptospirosis was associated with a seven-fold increase in risk for acquiring a leptospiral infection during prospective follow-up (OR 7.0, 95% CI, 3.2–15.7). The majority of significant risk factors were associated with the household environment. Subjects who resided in households that were situated in regions of the site with lower elevation (a proxy for flood risk) who lived in proximity to open sewers, vegetation and accumulated trash, and who had reported frequent rat sightings in the peridomicilary environment had increased risk for leptospiral infection. We also identified specific individual-level risk exposures in the household setting, such as reported contact with mud or trash, and cleaning a blocked sewer. Although we found occupation-associated exposures such as work related to garbage removal, sewers and construction as risk factors, the frequency of such factors was low among infected individuals (2–14%) and among overall community subjects (1–7%).

**Table 1:** Univariable risk factors for leptospiral infection duringduring prospective follow-up of the urban slum cohort, 2003–2007.

	Infection during follow-up event^1^ (N = 199)‏	No infection during follow-up event^1^ (N = 5427)	
Characteristics	No. or Median (% or IQR) ^2^		OR (95% CI)‏^3^
**Follow-up year**			
Year 1	51 (26%)	1534 (28%)	Ref
Year 2	26 (13%)	1298 (24%)	0.60 (0.37–0.97)
Year 3	27 (14%)	1367 (25%)	0.57 (0.37–0.89)
Year 4	95 (48%)	1228 (23%)	2.33 (1.68–3.23)
**Individual risk factors**			
Age (years)	26 (17.5–37)	24 (14–38)	1.01 (1.00–1.01)
Male gender	115 (58%)	2268 (42%)	1.94 (1.43–2.64)
Black ethnicity	77 (39%)	1953 (36%)	1.13 (0.83–1.54)
Illiteracy	54 (27%)	926 (17%)	1.82 (1.32–2.56)
Daily per capita household income (US$/day)	1.57 (0.66–2.79)	1.51 (0.78–2.83)	1.04 (0.98–1.10)
Informal employment	90 (45%)	1811 (33%)	1.59 (1.18–2.15)
History of prior hospitalization for leptospirosis	6 (3%)	25 (0%)	7.03 (3.15–15.71)
**Household-related environment**			
Household elevation (1m)	47.3 (36.1–61.2)	53.2 (40.7–62.8)	0.98 (0.97–0.99)
Distance from an open waste sewer (1m)	20.1 (7.3–36.9)	22.7 (9.0–40.2)	0.99 (0.99–1.00)
Vegetation within 10m of home	161 (81%)	3720 (69%)	1.96 (1.34–2.87)
Accumulated trash within 10m of home	182 (92%)	4504 (83%)	2.23 (1.33–3.74)
**Household-associated behavioral exposures**			
Digging in or cleaning blocked sewers	47 (24%)	752 (14%)	1.72 (1.20–2.46)
Contact with trash near home	56 (28%)	1040 (19%)	1.52 (1.08–2.15)
Contact with mud near home	95 (48%)	1978 (36%)	1.53 (1.15–2.05)
Contact with floodwater near home	81 (41%)	2326 (43%)	0.87 (0.64–1.17)
**Reservoir-related exposures**			
Reporting rats in the peridomicilary environment	157 (79%)	3710	1.63 (1.15–2.30)
Dogs in household	82 (41%)	2095 (39%)	1.08 (0.80–1.46)
**Occupation-related exposures**			
Work in construction	28 (14%)	356 (7%)	2.38 (1.56–3.64)
Work related to garbage removal	4 (2%)	17 (0%)	6.82 (1.99–23.38)
Work associated with contact to sewers	4 (2%)	56 (1%)	2.25 (0.85–5.99)

^**1**^N represents number of annual follow-up events (total, 5626)) with or without serologic evidence of infection.

^**2**^No^**2**^., number; IQR, interquartile range; Percentages reflect cases without missing values.

^3^OR, Odds Ratio; CI, Confidence Interval; Ref, reference.

After stepwise selection of fixed effects, we constructed a mixed model that included significant variables, together with spatiotemporal and individual-level random effects; the model with only the spatio-temporal random effects has DIC = 1700.58, whilst the model fitted with both spatio-temporal and individual-level random effects has DIC = 1698.13. Compared to the first year of follow-up, years 2 and 3 were associated with lower infection risk and year 4 had a higher risk (Table 2). Temporal differences in risk did not correlate with levels of pluviometric precipitation or number of extreme or heavy rainfall events that occurred during annual follow-up intervals. We found that male gender, age (peaking at 20 years of age, S1 Fig), and illiteracy were significant risk factors for infection (Table 2). Environmental factors and exposures related to topography and ground characteristics of the slum microenvironment were important determinants of infection risk. Peridomestic rodent infestation was a significant risk factor (OR 1.46, 95% CI 1.00–2.16). Contact with mud in the peridomicilary environment was a significant environmental exposure for infection (OR 1.57, 95% CI 1.13–2.17). Additionally there was a nearly significant linear relationship between higher household elevation and lower infection risk (OR 0.92, 95% CI 0.82–1.04 for each 10 meter increase in elevation.)

**Table 2:** Multivariable risk factors for leptospiral infection during prospective four-year follow-up of the urban slum cohort.

Characteristic	OR (95% CI)^1^
**Year of follow-up**	
Year 1	Ref
Year 2	0.64 (0.38–1.05)
Year 3	0.58 (0.35–0.95)
Year 4	2.45 (1.65–3.67)
**Male gender**	2.09 (1.54–2.84)
**Age**^2^	
Age (0–20 years old)	1.11 (1.06–1.17)
Age (>20 years old)	0.98 (0.97–1.00)
**Illiteracy**	1.88 (1.29–2.70)
**Household elevation (per 10m increase)**	0.92 (0.82–1.04)
**Peridomiciliary rat infestation**	1.46 (1.00–2.16)
**Peridomiciliary contact with mud**	1.57 (1.13–2.17)

^1^ OR, Odds Ratio; CI, Confidence Interval; Ref, reference.

^2^ The effect of age is modeled as a broken linear model with a transition at 20 years old, as informed by the relationship described by Generalized Additive Modeling (S1 Fig)

**Fig 3:**
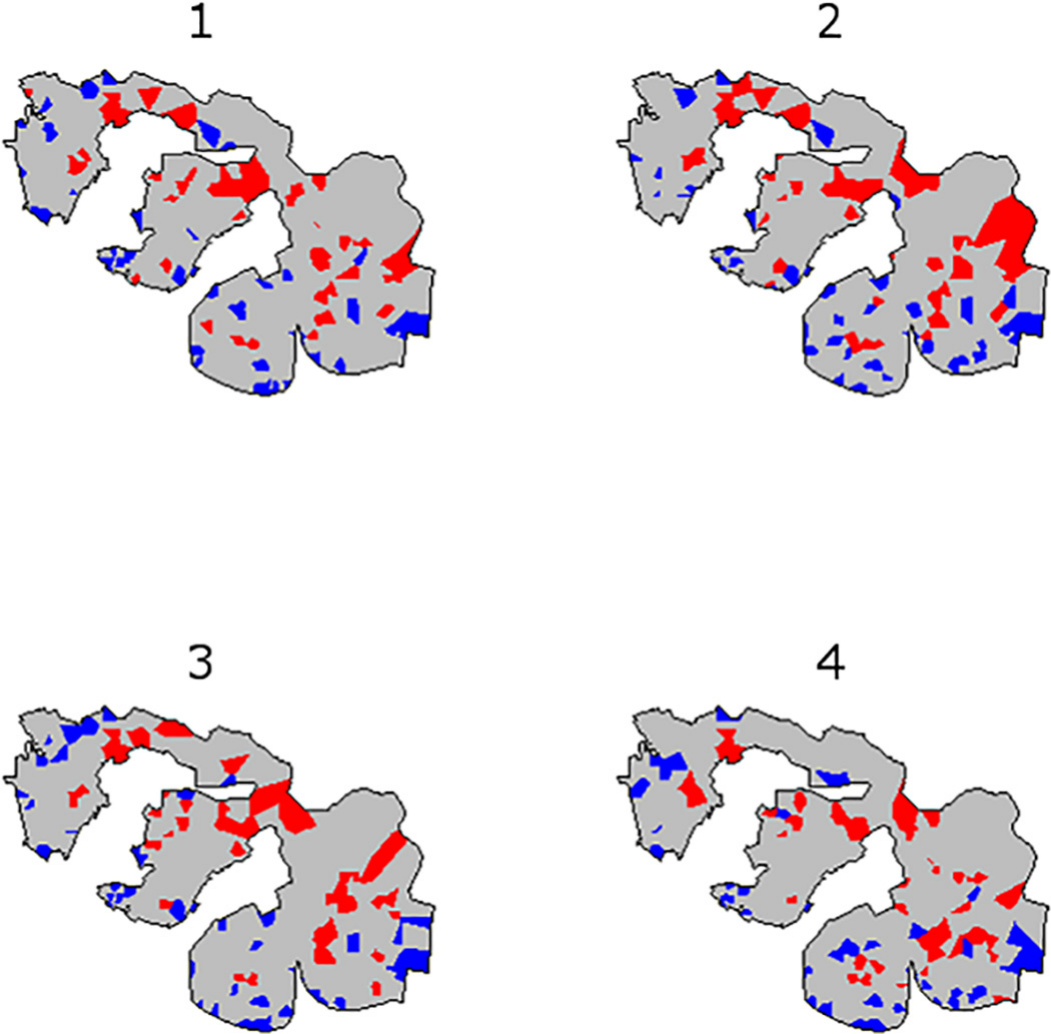
Risk of leptospiral infection within the Pau da Lima study site at each of four annual repeated measures during prospective follow-up of the community cohort. The choropleth map indicates the 50 areas with highest odds of infection (red) and the 50 areas with lowest odds of infection (blue) at each follow-up period.

We found that the spatial risk of infection was highly heterogeneous within the slum community. As seen in the choropleth maps in Fig 3, odds of infection varied significantly across regions of the study area. In general, “hot-spots” of increased infection risk were situated in valley bottoms and in the northern region with lowest elevation of the slum settlement, which were recently invaded by squatters, had more vegetation and had the least access to services such as formal or informal refuse collection (Fig 1). However, hot spots were frequently juxtaposed to “cold-spots” of infection risk by distances of less than 20–30 meters. Of note, the temporal distribution of regions of high and low infection risk remained relatively stable during the four annual follow-up periods, indicating that the same or similar risk exposures occur year to year in specific microenvironments within the slum community.

The fixed effects components of the model accounted for a large amount of the spatiotemporal heterogeneity in the leptospiral infection risk observed at the slum community site. The overall odds of infection in each follow-up period are plotted in the choropleth maps in S2A Fig. S2B Fig plots the fixed effects component of infection odds, indicating the spatial variation in infection risk that is explained by the variables included in the model (Table 2). Yet we also observed significant unexplained spatial and temporal variation in infection risk, which we accounted for in our modeling strategy through the random effect terms. S2C Fig plots the random effect component of infection odds, i.e. the component of the overall spatial variation in infection risk that could not be explained by the regression component of the model. Similar to the overall odds of infection, the spatial distribution of high and low risk areas associated with fixed effects (S2B Fig) and random effects (S2C Fig) remained similar year-to-year during prospective follow-up.

## Discussion

We present the results of a large prospective community-based study to determine rates and risks for *Leptospira* infection in an urban slum where leptospirosis is endemic. We followed a cohort of residents for four years and developed a spatiotemporal mixed model to identify risk factors and capture the distribution of unexplained variation in *Leptospira* infection. The model combines regression effects of explanatory variables, spatiotemporally correlated random effects, and uncorrelated individual-level random effects. Through these analyses, we found that *Leptospira* transmission in urban slums occurs due to the interaction of poverty, geography and climate. Our analysis of long-term prospective data confirms previous studies that identified socioeconomic and environmental risk factors but were limited by their retrospective design or limited follow-up [12,20–22,33]. Importantly, we found that among other mechanisms for environmental exposure, at-risk slum residents become infected with *Leptospira* through behaviors that lead to contact with contaminated soil and mud, mobilized most often by floodwater during heavy rain events. Individuals who live within an environment that is generally associated with elevated infection risk have specific characteristics and behaviors that elevate their risk of infection. In addition, we demonstrate that there exists a spatially stable, temporally varying layer of infection risk that is intrinsic to the environment on a fine scale, over and above the variation attributable to measured risk-factors. Risk for *Leptospira* infection in urban slums is therefore determined in large part by structural features, both social and environmental.

We found a high incidence of leptospiral infection (35.4 per 1,000 annual follow-up events) that disproportionately affected specific risk groups within our slum community site, which has an overall high level of absolute poverty. History of prior hospitalization for leptospirosis was associated with a markedly higher risk for serological conversion. We previously observed during one-year follow-up of this cohort [21] that serologic evidence for leptospiral infection occurred in individuals who had a positive baseline agglutinating antibody titer, presumably due to an exposure prior to the study. This investigation found that indeed, there were 22 repeat infections, including one tertiary infection, which occurred after cohort subjects acquired a documented initial infection during follow-up. These findings demonstrate that repeat exposure to the pathogenic bacteria is a frequent event among slum residents. To date, little is known about repeat leptospiral infections and the role of naturally-acquired immunity to reinfection due to the lack of population-based prospective studies. Our long-term cohort study suggests that an initial exposure, whether resulting in clinical disease or asymptomatic infection, confers at best partial immunity to a subsequent re-infection, in a setting where one predominant serovar is circulating.

We identified demographic and individual risk factors for infection that were consistent with previous studies in this population [12,21], as well as for leptospirosis in other epidemiologic settings [34,35]. Infection risk was highest in young adults and in males. Functional illiteracy, which is a marker of low social status and social exclusion, was also an important risk factor. In univariable analysis, garbage workers and construction workers also had significantly higher risk, possibly reflecting risk exposures related to social status as well as specific occupational exposure to mud and other environments contaminated by rat urine. These findings relating to the social determinants of infection risk indicate that behavior-related [33] differences influence the frequency or intensity of contact with contaminated environmental sources, leading to infection by *Leptospira*.

Within a slum community that is characterized by poor sanitation infrastructure and widespread rodent infestation, we found that distinct differences in the microenvironment lead to substantial spatial variation in infection risk. Lower household elevation was an environmental risk factor for infection in our model. In the study community, elevation correlates, in part, with socioeconomic gradient, with more impoverished squatters settling in regions of low elevation and poor land quality (Fig 1). Importantly, low elevation is also a proxy for risk of flooding, which typically occurs during periods of heavy rainfall in this and similar communities due to poor rainwater drainage infrastructure, leading to rapid accumulation of rainwater, mudslides, and overflow from open-air sewer canals. Flooding is known to promote leptospirosis transmission and is associated with both seasonal incidence fluctuation and outbreaks in the setting of extreme weather events [5,7,16,17,35,38–40].

Yet, we found a complex relationship between flooding and infection risk that sheds light on the environmental dynamic of *Leptospira* and the conditions that lead to human infection. As previously observed in this community [12,21], participants who report contact with mud had a significantly higher risk of infection. However, reported contact with floodwater was not found to be an independent risk factor. At low elevations, the majority of ground surfaces are unpaved dirt, leading to mobilization of mud, and risk of landslides during heavy rain. In contrast, participants who reported contact with floodwater without mud were more likely to live at higher elevation, where flooding reflects poor rainwater drainage of asphalted surfaces. Our analyses reveal the important role of the peridomiciliary environment in facilitating infectious contact with environmentally transmitted pathogens such as *Leptospira*. While survival of *L*. *interrogans* in soil and water of urban slums is unknown, studies in other settings have proved that this organism can survive from days to months in the environment [41]. In this case, soil may serve as an important environmental reservoir for pathogenic *Leptospira* in urban environments with heavy rat infestation and poor infrastructure. Heavy rainfall may also increase disease transmission by mobilizing the pathogen from this reservoir and promoting contact of slum residents with contaminated environmental sources at the soil-water interface.

There was substantial temporal variation in infection risk. During the fourth follow-up period, the risk of infection was 3.1 times higher than in earlier years (95% CI for RR, 2.4–4.1). We found similar risk factors for infection during this year compared to the preceding three years, and a similar spatial distribution (Fig 3). While this study was not structured to understand the specific determinants of temporal variation in incidence, there were no unusual patterns of rainfall, humidity, or temperature in follow-up year 4 compared to preceding years. This high incidence in asymptomatic transmission in year 4 may have occurred due to stochastic differences in rodent density within the community, changes in flooding patterns related to upstream changes in rainwater drainage infrastructure, or other unmeasured factors leading to more frequent exposure. Temporal fluctuation in asymptomatic *Leptospira* infection has not previously been studied. There were no detected changes in the incidence of severe leptospirosis during year 4, but it is not known whether there was a concomitant fluctuation in nonspecific febrile illness. However, these findings provide support for the importance of including adjustment for temporal variability when attempting to understand the risk factors of infection in complex ecological systems such as urban slums, and provide important information to understand the natural history of urban leptospirosis.

In contrast to the marked temporal variation we observed, the spatial distribution of risk was relatively constant from year to year. The fixed effects in the model account for much of this spatial variation in risk, but there was also a substantial residual component of risk that was best explained by the spatial random effect. By plotting this unexplained risk, discrete locations within the study area were identified with markedly higher or lower risk than explained by the fixed effects in the model. Based on these analyses, there may be localities within the study area that have specific unique characteristics, not captured by the fixed factors included in our multivariable regression, which influence risk of infection. These areas should be further investigated for possible explanatory characteristics that may lead to identification of novel hypotheses about disease transmission mechanisms in this community, and possible opportunities for targeted, informed intervention to decrease the burden of this infection.

There are limitations to this study. Among individuals who were lost to follow-up, incidence was inversely correlated with the number of years of completed follow-up, suggesting a potential source of bias. The estimate in our random effects model is the incidence that would apply in the absence of loss to follow-up. Compared to those with complete follow-up, people who participated in the study but were lost to complete follow-up had similar income but were more likely to be male, an important risk category for infection, thus the true overall incidence in this community may be higher than our results indicate.

This study was designed to understand the individual and environmental risk factors for disease transmission while adjusting for temporal variability in infection risk, but did not aim to identify specific explanatory variables that explain annual incidence fluctuation. We therefore did not include any temporally varying data in the model, such as precipitation, temperature, humidity, or ultraviolet light intensity, which might influence the frequency of flooding events or the environmental survival of *Leptospira*. However, based on our model, the spatial pattern of risk was relatively constant over time. This suggests that our model adequately adjusted for temporal variation by including follow-up event as an independent fixed effect without interaction with other variables. Future studies should include time-varying data in order to provide improved explanatory information about annual fluctuations in infection rates.

Although leptospirosis incidence in Brazil and other settings fluctuates seasonally in close association with rainfall patterns [5,17,38], nothing is known about the effects of seasonal weather differences on the temporal dynamics of asymptomatic infection. Studies that aim to understand the dynamic of *Leptospira* transmission during rainy and dry seasons would be valuable to understand the impact of environmental factors on the development of severe disease. Furthermore, the population dynamics of the rat population and shedding of the leptospiral pathogen from the reservoir may influence the spatio-temporal distribution of risk in the urban slum setting. Recent studies suggest that the demography, movement patterns and *Leptospira* shedding from *R*. *norvegicus*, the main reservoir in the study area, may influence the spatial dynamics of human infection risk but perhaps not the temporality [14,23,36,37]. However future work needs to be performed that combine ecological and epidemiological studies to establish the spatiotemporal link between reservoir transmission and risk of spill-over infection to humans.

Despite these limitations, we performed rigorous adjustment for spatial and temporal variation in disease transmission risk, and therefore are able to draw important conclusions about the drivers of leptospirosis infection in high-risk urban slum environments. We demonstrate that there exist specific risk groups of individuals, including young adults, males, and individuals with marginalized social status and high-risk occupational conditions, who may have activities that place them in more frequent or more intense contact with contaminated environmental sources of transmission. In addition, our analyses suggest that mobilization of soil and mud contaminated by infected rodent urine may be an important process leading to human exposure to pathogenic *Leptospira*. This draws attention to the potentially important role of exposed soil as an environmental reservoir for pathogenic *Leptospira*. Eco-epidemiological studies are therefore needed to provide an integrated understanding of the dynamic of *Leptospira* transmission between the rodent reservoir, soil and other environmental compartments, and human hosts. Finally, our findings suggest that structural interventions may be capable of reducing the burden of leptospirosis in this and other vulnerable communities by protecting residents from contact with contaminated soil and mud during heavy rain events.

## Supporting Information

S1 Checklist STROBE Checklist. (DOC)

S1 Fig Relationship between age and leptospiral infection obtained from the fit of a generalized additive model (GAM) adjusting for follow-up year, male gender, literacy, household elevation, observation of rats near home, and contact with mud near the household. (TIFF)

S2 FigRisk of leptospiral infection within the Pau da Lima study site at each of four annual repeated measures during prospective follow-up of the community cohort.Choropleth maps were constructed to represent the spatial distribution of infection risk, and the components of that observed risk as described by our multivariable mixed effects model for log odds: $\log\;\left(\frac{p_{ij}}{1-p_{ij}}\right)=z_{ij}\beta +S(i,j)+u_{i}$, where for individual *i* at time *j*, *p*_*ij*_ represents the probability of infection, *z*_*ij*_ denotes the vector of covariates, *β* represents the coefficient factor, *S*(*i*,*j*) are spatio-temporal random effects and u_i_ are uncorrelated individual-level random effects. In S2A Fig, the odds of infection, including the random effects component, are therefore represented as $\frac{p_{ij}}{1-p_{ij}}=\exp\left(z_{ij}\beta \right)\times exp(S(i,j)+u_{i})$. In S2B Fig, we plot only the fixed effects component of odds: exp(*z*_*ij*_*β*). In S2C Fig, we plot only the random effects component of odds: exp(*S*(*i*,*j*) + *u*_*i*_).(TIF)

S1 Table Characteristics of 2,003 cohort subjects according to duration of prospective follow-up at the urban slum study site from 2003–2007. (DOCX)

S2 Table Crude and age- and gender-specific leptospiral infection rates at the Pau da Lima study site, 2003–2007. (DOCX)

S1 File Anonymized dataset including four repeated annual measurements of anti-Leptospira serology and risk factors in an urban slum cohort. (CSV)

S2 File Data dictionary for S1 File. (DOC)
