# Supplementary material for: Spatiotemporal Determinants of Urban Leptospirosis Transmission: Four-Year Prospective Cohort Study of Slum Residents in Brazil
Source: PLoS Negl Trop Dis. 2016 Jan 15;10(1):e0004275. doi: 10.1371/journal.pntd.0004275 (PMC4714915; doi:10.1371/journal.pntd.0004275)
Supplement: S1 Checklist — (DOC) [file pntd.0004275.s001.doc]

STROBE Statement—Checklist of items that should be included in reports of ***cohort studies***

|  | Item No | Recommendation |
| --- | --- | --- |
| **Title and abstract** | 1 | (*a*) Indicate the study’s design with a commonly used term in the title or the abstract  Yes: pages 1, 3 |
|  |  | (*b*) Provide in the abstract an informative and balanced summary of what was done and what was found  Yes: page 3 |
| Introduction | | |
| Background/rationale | 2 | Explain the scientific background and rationale for the investigation being reported  Yes: pages 6-7 |
| Objectives | 3 | State specific objectives, including any prespecified hypotheses  Yes: page 7 |
| Methods | | |
| Study design | 4 | Present key elements of study design early in the paper  Yes: pages 8-10 |
| Setting | 5 | Describe the setting, locations, and relevant dates, including periods of recruitment, exposure, follow-up, and data collection  Yes: pages 8-10 |
| Participants | 6 | (*a*) Give the eligibility criteria, and the sources and methods of selection of participants. Describe methods of follow-up  Yes: page 8-10 |
|  |  | (*b*) For matched studies, give matching criteria and number of exposed and unexposed  NA |
| Variables | 7 | Clearly define all outcomes, exposures, predictors, potential confounders, and effect modifiers. Give diagnostic criteria, if applicable  Yes: pages 9-11 |
| Data sources/ measurement | 8* | For each variable of interest, give sources of data and details of methods of assessment (measurement). Describe comparability of assessment methods if there is more than one group  Yes: pages 9-10 |
| Bias | 9 | Describe any efforts to address potential sources of bias  Yes: pages 9-10 |
| Study size | 10 | Explain how the study size was arrived at  Yes: pages 8 |
| Quantitative variables | 11 | Explain how quantitative variables were handled in the analyses. If applicable, describe which groupings were chosen and why  Yes: pages 10-11 |
| Statistical methods | 12 | (*a*) Describe all statistical methods, including those used to control for confounding  Yes: pages 10-11 |
|  |  | (*b*) Describe any methods used to examine subgroups and interactions  Yes: page 11 |
|  |  | (*c*) Explain how missing data were addressed  Yes: page 10 |
|  |  | (*d*) If applicable, explain how loss to follow-up was addressed  Yes: page 12 |
|  |  | (*e*) Describe any sensitivity analyses  NA |
| Results | | |
| Participants | 13* | (a) Report numbers of individuals at each stage of study—eg numbers potentially eligible, examined for eligibility, confirmed eligible, included in the study, completing follow-up, and analysed  Yes: page 12, S1 Table |
|  |  | (b) Give reasons for non-participation at each stage  Yes: page 12 |
|  |  | (c) Consider use of a flow diagram  NA |
| Descriptive data | 14* | (a) Give characteristics of study participants (eg demographic, clinical, social) and information on exposures and potential confounders  Yes: page 12, S1 Table |
|  |  | (b) Indicate number of participants with missing data for each variable of interest  Yes: S1 Table |
|  |  | (c) Summarise follow-up time (eg, average and total amount)  Yes: page 12, S1 Table |
| Outcome data | 15* | Report numbers of outcome events or summary measures over time  Yes: S2 Table |
| Main results | 16 | (*a*) Give unadjusted estimates and, if applicable, confounder-adjusted estimates and their precision (eg, 95% confidence interval). Make clear which confounders were adjusted for and why they were included  Yes: Tables 1-2 |
|  |  | (*b*) Report category boundaries when continuous variables were categorized  Yes: Tables 1-2 |
|  |  | (*c*) If relevant, consider translating estimates of relative risk into absolute risk for a meaningful time period  NA |
| Other analyses | 17 | Report other analyses done—eg analyses of subgroups and interactions, and sensitivity analyses  Yes: Figure S1, Figure S2, Figure 2-3, Table S2 |
| Discussion | | |
| Key results | 18 | Summarise key results with reference to study objectives  Yes: pages 12-14 |
| Limitations | 19 | Discuss limitations of the study, taking into account sources of potential bias or imprecision. Discuss both direction and magnitude of any potential bias  Yes: pages 18 |
| Interpretation | 20 | Give a cautious overall interpretation of results considering objectives, limitations, multiplicity of analyses, results from similar studies, and other relevant evidence  Yes: pages 14-17, 19 |
| Generalisability | 21 | Discuss the generalisability (external validity) of the study results  Yes: page 16 |
| Other information | | |
| Funding | 22 | Give the source of funding and the role of the funders for the present study and, if applicable, for the original study on which the present article is based  Yes |

*Give information separately for exposed and unexposed groups.

**Note:** An Explanation and Elaboration article discusses each checklist item and gives methodological background and published examples of transparent reporting. The STROBE checklist is best used in conjunction with this article (freely available on the Web sites of PLoS Medicine at http://www.plosmedicine.org/, Annals of Internal Medicine at http://www.annals.org/, and Epidemiology at http://www.epidem.com/). Information on the STROBE Initiative is available at http://www.strobe-statement.org.
