# Supplementary figures and images for: Spatiotemporal Determinants of Urban Leptospirosis Transmission: Four-Year Prospective Cohort Study of Slum Residents in Brazil
Source: PLoS Negl Trop Dis. 2016 Jan 15;10(1):e0004275. doi: 10.1371/journal.pntd.0004275 (PMC4714915; doi:10.1371/journal.pntd.0004275)

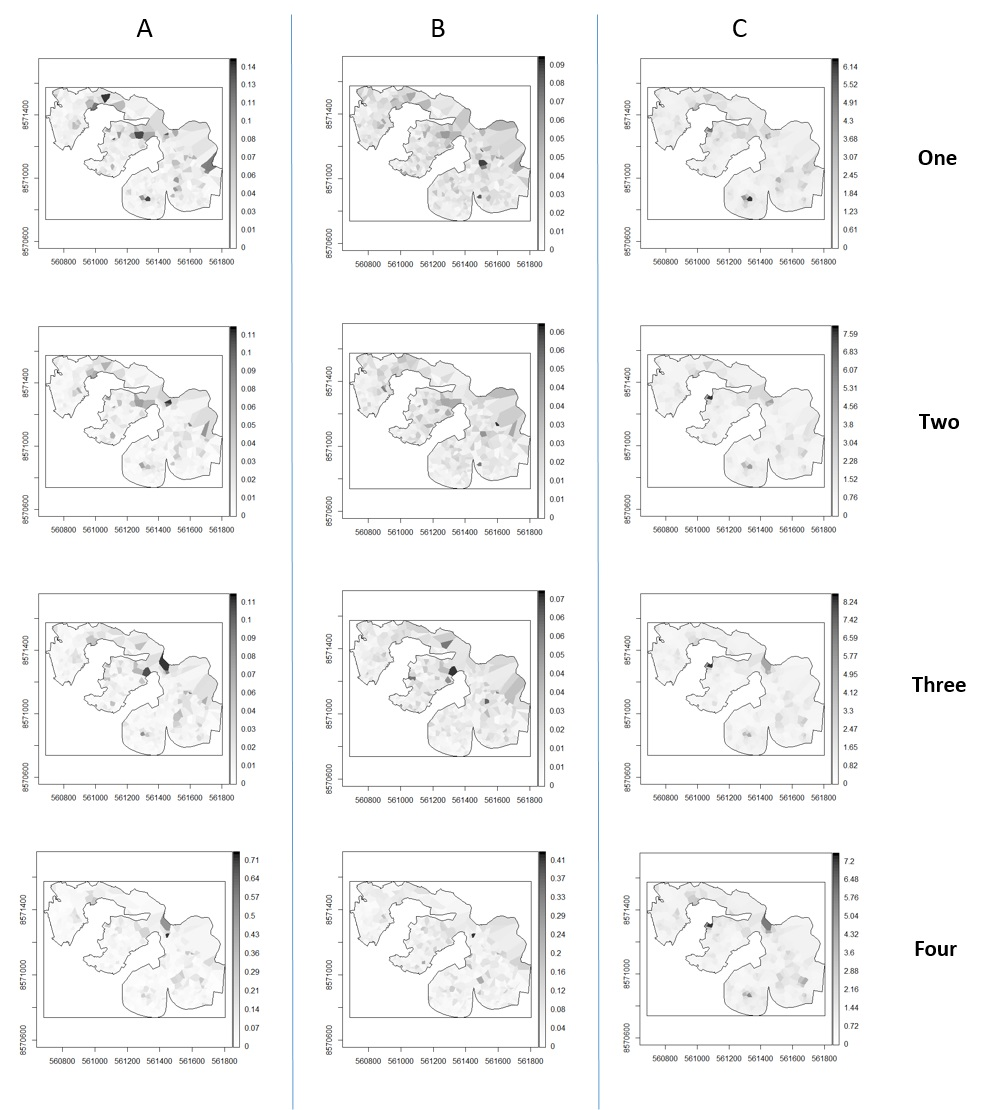

Supplement: S2 Fig — Choropleth maps were constructed to represent the spatial distribution of infection risk, and the components of that observed risk as described by our multivariable mixed effects model for log odds: log(pij1−pij)=zijβ+S(i,j)+ui, where for individual i at time j, pij represents the probability of infection, zij denotes the vector of covariates, β represents the coefficient factor, S(i,j) are spatio-temporal random effects and ui are uncorrelated individual-level random effects. In S2A Fig, the odds of infection, including the random effects component, are therefore represented as pij1−pij=exp(zijβ)×exp(S(i,j)+ui). In S2B Fig, we plot only the fixed effects component of odds: exp(zijβ). In S2C Fig, we plot only the random effects component of odds: exp(S(i,j) + ui). (TIF) [file pntd.0004275.s003.tif]
