# Supplementary material for: Spatiotemporal Determinants of Urban Leptospirosis Transmission: Four-Year Prospective Cohort Study of Slum Residents in Brazil
Source: PLoS Negl Trop Dis. 2016 Jan 15;10(1):e0004275. doi: 10.1371/journal.pntd.0004275 (PMC4714915; doi:10.1371/journal.pntd.0004275)
Supplement: S1 Table — (DOCX) [file pntd.0004275.s004.docx]

**S1 Table.** Characteristics of 2,003 cohort subjects according to duration of prospective follow-up at the urban slum study site from 2003-2007.

| **Characteristics** | **Duration of follow up** | | | | | **P-value^1^** |
| --- | --- | --- | --- | --- | --- | --- |
|  | **4 years** (N=1127)‏ | **3 years** (N=160) | **2 years** (N=195) | **1 year** (N=248) | **0 years** (N=273) |  |
| Age | 23 (12–37) | 22 (17–34) | 23 (16–36) | 24 (17–35) | 23 (17–32) | 0.076^2^ |
| Male gender | 468 (42%) | 63 (39%) | 100 (51%) | 122 (49%) | 127 (47%) | 0.014^3^ |
| Black race | 405 (36%) | 54 (34%) | 80 (41%) | 88 (36%) | 66 (24%) | 0.153^3^ |
| Daily per capita household income (dollar/day) | 0.79 (0.28–1.26) | 0.79 (0.30–1.55) | 0.92 (0.50–1.46) | 0.73 (0.23–1.23) | 0.90 (0.37–1.69) | 0.029^2^ |
| Mean incidence (infections/1000 follow up events) | 36.4 (31.1–42.3) | 20.8 (10.7–37.0) | 38.5 (22.5–61.8) | 40.3 (20.7–71.5) | N/A | 0.335^4^ |

N=Number of patients; IQR = Inter-quartile range; NA=Not applicable; ^1^P-value comparing complete follow up vs. any loss to follow up.  ^2^Kruskal-Wallas test. ^3^Chi-square test. ^4^Chi-square test, including participants with at least one year of follow up.
