# Supplementary material for: Spatiotemporal Determinants of Urban Leptospirosis Transmission: Four-Year Prospective Cohort Study of Slum Residents in Brazil
Source: PLoS Negl Trop Dis. 2016 Jan 15;10(1):e0004275. doi: 10.1371/journal.pntd.0004275 (PMC4714915; doi:10.1371/journal.pntd.0004275)
Supplement: S2 Table — (DOCX) [file pntd.0004275.s005.docx]

**S2 Table**. Crude and age- and gender-specific leptospiral infection rates at the Pau da Lima study site, 2003-2007.

|  |  | **Total** |  |  | **Male** |  |  | **Female** |  |
| --- | --- | --- | --- | --- | --- | --- | --- | --- | --- |
|  | No. infections  (Follow-up events) | Rate | 95% CI | No. infections  (Follow-up events) | Rate | 95% CI | No. infections  (Follow-up events) | Rate | 95% CI |
| Follow-up one |  |  |  |  |  |  |  |  |  |
| 5-14 years | 12 (441) | 27.21 | 14.86 - 46.09 | 9 (218) | 41.28 | 20.39 - 75.35 | 3 (223) | 13.45 | 3.72 - 35.89 |
| 15-24 years | 17 (410) | 41.46 | 25.07 - 64.89 | 12 (171) | 70.18 | 38.32 - 118.86 | 5 (239) | 20.92 | 7.93 - 45.86 |
| 25-34 years | 14 (278) | 50.36 | 28.84 - 82.24 | 7 (114) | 61.4 | 27.38 - 120.57 | 7 (164) | 42.68 | 19.03 - 83.81 |
| 35-44 years | 7 (223) | 31.39 | 14.00 - 61.64 | 2 (81) | 24.69 | 4.92 - 79.15 | 5 (142) | 35.21 | 13.35 - 77.18 |
| >44 years | 1 (233) | 4.29 | 0.39 - 20.01 | 1 (86) | 11.63 | 1.05 - 54.21 | 0 (147) | 0.00 | 0.00 - 16.76 |
| All | 51 (1585) | 32.18 | 24.23 - 41.95 | 31 (670) | 46.27 | 32.05 - 64.8 | 20 (915) | 21.86 | 13.77 - 33.09 |
| Follow-up two |  |  |  |  |  |  |  |  |  |
| 5-14 years | 3 (350) | 8.57 | 2.37 - 22.87 | 3 (174) | 17.24 | 4.77–46.00 | 0 (176) | 0.00 | 0 .00– 14.00 |
| 15-24 years | 6 (340) | 17.65 | 7.34 - 36.38 | 4 (149) | 26.85 | 8.98 - 63.83 | 2 (191) | 10.47 | 2.09 - 33.56 |
| 25-34 years | 6 (222) | 27.03 | 11.23 - 55.71 | 3 (83) | 36.14 | 10.00 - 96.43 | 3 (139) | 21.58 | 5.97 - 57.58 |
| 35-44 years | 5 (201) | 24.88 | 9.43 - 54.53 | 3 (66) | 45.45 | 12.58 - 121.27 | 2 (135) | 14.81 | 2.95 - 47.49 |
| >44 years | 6 (211) | 28.44 | 11.82 - 58.62 | 4 (72) | 55.56 | 18.57 - 132.08 | 2 (139) | 14.39 | 2.87 - 46.12 |
| All | 26 (1324) | 19.64 | 13.13 - 28.33 | 17 (544) | 31.25 | 18.90 - 48.90 | 9 (780) | 11.54 | 5.70 - 21.06 |
| Follow-up three |  |  |  |  |  |  |  |  |  |
| 5-14 years | 3 (330) | 9.09 | 2.52 - 24.25 | 1 (159) | 6.29 | 0.57 - 29.32 | 2 (171) | 11.70 | 2.33 - 37.49 |
| 15-24 years | 6 (359) | 16.71 | 6.95 - 34.45 | 3 (168) | 17.86 | 4.94 - 47.64 | 3 (191) | 15.71 | 4.35 - 41.90 |
| 25-34 years | 11 (250) | 44 | 23.35 - 76.16 | 7 (99) | 70.71 | 31.53 - 138.83 | 4 (151) | 26.49 | 8.86 - 62.98 |
| 35-44 years | 2 (217) | 9.22 | 1.84 - 29.54 | 1 (81) | 12.35 | 1.12 - 57.56 | 1 (136) | 7.35 | 0.67 - 34.28 |
| >44 years | 5 (238) | 21.01 | 7.97 - 46.05 | 2 (86) | 23.26 | 4.64 - 74.54 | 3 (152) | 19.74 | 5.46 - 52.66 |
| All | 27 (1394) | 19.37 | 13.05 - 27.76 | 14 (593) | 23.61 | 13.52 - 38.55 | 13 (801) | 16.23 | 9.09 - 26.96 |
| Follow-up four |  |  |  |  |  |  |  |  |  |
| 5-14 years | 16 (296) | 54.05 | 32.15 - 85.69 | 11 (145) | 75.86 | 40.26 - 131.3 | 5 (151) | 33.11 | 12.56 - 72.58 |
| 15-24 years | 27 (323) | 83.59 | 56.33 - 119.79 | 20 (162) | 123.46 | 77.79 - 186.92 | 7 (161) | 43.48 | 19.39 - 85.37 |
| 25-34 years | 21 (251) | 83.67 | 53.34 - 125.48 | 8 (97) | 82.47 | 38.90 - 155.63 | 13 (154) | 84.42 | 47.27 - 140.25 |
| 35-44 years | 15 (217) | 69.12 | 40.38 - 111.14 | 7 (82) | 85.37 | 38.07 - 167.62 | 8 (135) | 59.26 | 27.95 - 111.82 |
| >44 years | 16 (236) | 67.8 | 40.33 - 107.47 | 7 (90) | 77.78 | 34.68 - 152.72 | 9 (146) | 61.64 | 30.45 - 112.51 |
| All | 95 (1323) | 71.81 | 58.43 - 87.36 | 53 (576) | 92.01 | 69.67 - 119.37 | 42 (747) | 56.22 | 41.09 - 75.23 |
| Overall |  |  |  |  |  |  |  |  |  |
| 5-14 years | 34 (1417) | 23.99 | 16.91 - 33.12 | 24 (696) | 34.48 | 22.66 - 50.45 | 10 (721) | 13.87 | 7.12 - 24.61 |
| 15-24 years | 56 (1432) | 39.11 | 29.84 - 50.39 | 39 (650) | 60.00 | 43.31 - 81.14 | 17 (782) | 21.74 | 13.14 - 34.02 |
| 25-34 years | 52 (1001) | 51.95 | 39.23 - 67.56 | 25 (393) | 63.61 | 42.18 - 92.39 | 27 (608) | 44.41 | 29.93 - 63.64 |
| 35-44 years | 29 (858) | 33.8 | 23.11 - 47.86 | 13 (310) | 41.94 | 23.48 - 69.67 | 16 (548) | 29.2 | 17.37 - 46.28 |
| >44 years | 28 (918) | 30.5 | 20.71 - 43.44 | 14 (334) | 41.92 | 24.00 - 68.45 | 14 (584) | 23.97 | 13.73 - 39.15 |
| All | 199 (5626) | 35.37 | 30.71 - 40.55 | 115 (2383) | 48.26 | 40.03 - 57.70 | 84 (3243) | 25.9 | 20.80 - 31.90 |

No, Number of infections; 95% CI, 95% Confidence Intervals.
Rates calculated per 1,000 annual follow-up events.
