# Supplementary material for: Spatiotemporal Determinants of Urban Leptospirosis Transmission: Four-Year Prospective Cohort Study of Slum Residents in Brazil
Source: PLoS Negl Trop Dis. 2016 Jan 15;10(1):e0004275. doi: 10.1371/journal.pntd.0004275 (PMC4714915; doi:10.1371/journal.pntd.0004275)
Supplement: S2 File — (DOC) [file pntd.0004275.s007.doc]

| **Variable** | **Definition** | | **Coding** |
| --- | --- | --- | --- |
| IDNO16 | Individual number | | (House number . resident number) |
| CASANO | Household number | | number |
| YEAR | Year of follow-up | | number |
| AGE | Age | | continuous |
| SEXFIM | Gender | | 0=F 1=M |
| RACARE | Declared race | | 0=white 1=”parda” 2=black 3=other 8= NSA |
| BLACK | Black race | | 0 = No 1 = Yes |
| ESTUDO | Highest level of education | | 0 =No schooling  1 = Incomplete elementary 2 = complete elementary 3 = incomplete highschool  4 = complete highschool 5 = Any university |
| FRASE | Literate | | 0 = No 1 = Yes |
| ESTUD | Subject was in school during the month of the interview | | 0 = No 1 = Yes |
| APOS | Retired? | | 0 = No 1 = Yes |
| DCAS | Housewife? | | 0 = No 1 = Yes |
| ALUG | House is rented? | | 0= No 1=Yes |
| MC | Duration in years individual has lived in current home | | Continuous years |
| REND | Total personal monthly income in Reais | | Continuous Reais |
| RENDUSS | Per capita household daily income in US$ | | Continuous dollars |
| IDENT | Participant has an ID card | | 0 = No 1 = Yes |
| CPF | Participant has a financial number | | 0 = No 1 = Yes |
| TIT | Participant owns the title to the household | | 0 = No 1 = Yes |
| CCART | Participant is employed with contract and benefits | | 0 = No 1 = Yes |
| SCART | Participant is employed without contract and benefits (participant may have second employment with benefits) | | 0 = No 1 = Yes |
| DEN | Diagnosed with dengue since last visit | | 0 = No 1 = Yes |
| VACFA | Vaccinated against yellow fever? | | 0 = No 1 = Yes |
| PASTLEPTO | Participant relates having had prior leptospirosis infection | | 0 = No 1 = Yes |
| ANTES | Participant reports having leptospirosis since last collection | | 0 = No 1 = Yes 8 = N/A 9 = does not know |
| CAANT | Household member wih lepto since last collection? | | 0 = No 1 = Yes 9 = don’t know |
| **Environmental variables** | | |  |
| ESTE | GIS coordinate east of household | | Number |
| NORTE | GIS coordinate north of household | | Number |
| HEIGHT | Elevation of household | | Meters |
| COTA | GIS distance to lowest point in the valley (meters) | | Meters |
| WASTE | GIS distance to nearest waste sewer (meters) | | Meters |
| ANYSEWER | GIS distance to nearest water drainage of any kind (meters) | | Meters |
| TRASH | GIS distance to nearest trash dump | | Meters |
| WASTECOTA | Distance to waste sewer and distance to lowest valley point | | 0: >=20m from both  1: |
| VALE | Valley number of the household | | number |
| **III.D Household environmental variables (from interview)** | |  | |
| ALACA | Household floods? | | 0 = No 1 = Yes |
| LXCA | Accumulated trash within 10m of house? | | 0 = No 1 = Yes |
| LXCN | Accumulated construction materials within 10m of house? | | 0 = No 1 = Yes |
| LXMT | Unused material within 10m of house? | | 0 = No 1 = Yes |
| VEG | Vegetation within 10m of house? | | 0 = No 1 = Yes |
| BANA | Banana tree within 10m of house? | | 0= No 1=Yes |
| **Behaviors** | |  | |
| CAALAD | Contact with flood water near house | | 0 = No 1 = Yes |
| CAESGD | Contact with sewer water near house | | 0 = No 1 = Yes |
| CLAMD | Contact with m ud near house | | 0 = No 1 = Yes |
| CLXD | Contact with trash near house | | 0 = No 1 = Yes |
| ESGCAVD | Participant was digging in sewer near house | | 0 = No 1 = Yes |
| ESGLIXD | Removed trash from sewer near house | | 0 = No 1 = Yes |
| ESGRUA | Unblocked the street sewer | | 0 = No 1 = Yes |
| **III.F Presença de Reservatórios no Ambiente Domiciliar** | |  | |
| RATD | Observation of rats near house | | 0 = No 1 = Yes |
| QRATD | Observed rats during the day near house | | 0 = No 1 = Yes |
| RD | Number of rats observed at a single time | | Number |
| CACH | Dog in household | | 0 = No 1 = Yes |
| GALC | Raise chickens | | 0 = No 1 = Yes |
| GAT | Cat in household | | 0 = No 1 = Yes |
| **Occupational** | |  | |
| TRBAI | Works in Pau da lima | | 0 = No 1 = Yes |
| GARI | Works in garbage removal | | 0 = No 1 = Yes |
| MECAN | Mechanic | | 0 = No 1 = Yes |
| PEDR | Construction | | 0 = No 1 = Yes |
| PRESG | Works with sewers | | 0 = No 1 = Yes |
| VAMB | Ambulatory vendor | | 0 = No 1 = Yes |
| PROUTR | Other job | | 0 = No 1 = Yes |
| **III.H Comportamento de Risco no Ambiente de Trabalho** | |  | |
| CAALAT | Contact with flood water near job | | 0 = No 1 = Yes |
| CAESGT | Contact with sewer water near job | | 0 = No 1 = Yes |
| CLAMT | Contact with mud near job | | 0 = No 1 = Yes |
| CLXT | Contact with trash near job | | 0 = No 1 = Yes |
| **III.I Reservoirs near work** | |  | |
| RATT | Observe rats near job | | 0 = No 1 = Yes |
| QRATT | Rats during the day at work | | 0 = No 1 = Yes |
| RT | Number of rats seen at a single time at work | | number |
| **Outcomes** |  | |  |
| **IV.A Infecção** |  | |  |
| Revinfec | Infection with leptospira | | 0 = No 1 = Yes |
